# Recent Advances in Hydrogel-Based Biosensors for Cancer Detection

**DOI:** 10.1021/acsami.4c02317

**Published:** 2024-08-27

**Authors:** Shengwei Sun, Jinju Chen

**Affiliations:** Department of Materials, Loughborough University, Loughborough LE11 3TU, United Kingdom

**Keywords:** hydrogel, biosensor, biomarker, fabrication, cancer detection, challenge

## Abstract

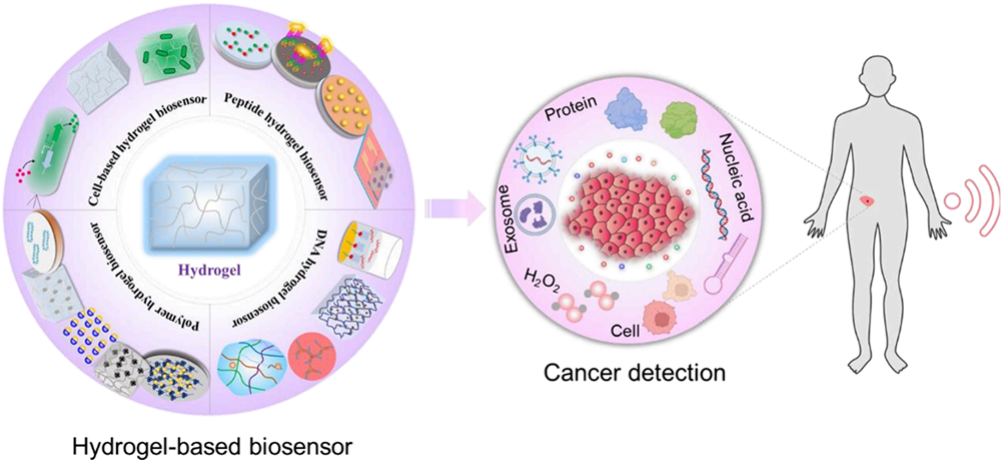

Early cancer detection is crucial for effective treatment, but current methods have limitations. Novel biomaterials, such as hydrogels, offer promising alternatives for developing biosensors for cancer detection. Hydrogels are three-dimensional and cross-linked networks of hydrophilic polymers that have properties similar to biological tissues. They can be combined with various biosensors to achieve high sensitivity, specificity, and stability. This review summarizes the recent advances in hydrogel-based biosensors for cancer detection, their synthesis, their applications, and their challenges. It also discusses the implications and future directions of this emerging field.

## 1 Introduction

Cancer is a major global health challenge, with an estimated 10 million deaths in 2020 alone.^1^ It is the second leading cause of death worldwide after cardiovascular diseases, accounting for nearly one in six deaths.^2^ Early detection, diagnosis, and treatment of cancer can significantly improve the survival and quality of life of cancer patients.^3^ For example, women who are diagnosed with cervical cancer before the cancer spread have a 5-year survival rate of 92%, compared with 15% for women who are diagnosed with the cancer at later stages (Jupiter Medical Center). Therefore, it is essential to detect cancer or precancerous change as early as possible, not only to identify the cancer itself but also to monitor the changes in its precursors or biomarkers.^4,5^ However, the current methods of cancer diagnosis, such as computed tomography (CT) scanning, nuclear magnetic resonance imaging (MRI), and positron emission tomography (PET) scanning are generally expensive, time-consuming, and lack the resolution to detect ultrasmall tumor cells at the early stage.^6^ Therefore, there is an urgent need for an economical, rapid, and early cancer monitoring and diagnostic approach with high selectivity, high sensitivity, low limit of detection (LOD), and broad dynamic range.^7^

Recent advances in bioanalytical techniques have led to the development and applications of biosensors as point-of-care/diagnostic devices.^8,9^ Biosensors are widely used in biomedical diagnosis and other fields, relying on biological/biochemical reactions mediated by tissues, the immune systems, enzymes, or whole cells to detect target analytes, with the output being a specific electrical, optical, or thermal signal.^10^ Through efficient biorecognition and signal transduction, they offer great potential for the detection of biologically relevant targets in a more rapid, accurate, and cost-effective manner, showing remarkable advantages over classical methods such as PCR, ELISA, mass spectrometry, and flow cytometry.^11^ So far, numerous biosensor devices have been designed with different functions to detect enzymes, proteins, antibodies, antigens, DNA, and exosomes in real human samples.^12−15^ All these biomolecules serving as potential disease markers have brought biosensors of great interest in shifting the medical paradigm from treatment to prevention.^16,17^

With the increasing demand for miniaturization, high-sensing efficiency, wide applicability, and cost reduction, the integration of high-performance biosensing platforms with hydrogels has become a significant trend, which represents a class of promising functional materials consisting of three-dimensional (3D) network structures produced by physical or chemical cross-linking.^18,19^ Hydrogels have been widely used as reliable biomaterials for the fabrication of medical devices and scaffold-based tissue engineering because of their unique properties. Since the first hydrogel was synthesized in 1960, a number of synthetic polymers and natural polymers have been pervasively used to produce hydrogels.^20^ Generally, the molecular units of these polymer chains shape the specific properties of the hydrogel, which enables further chemical modification to construct synthetic networks with tunable functional properties.^18,21^ Moreover, hydrogels can be designed to respond to various stimuli, such as temperature, pH, light, ionic changes, and redox potential, by exploiting the physicochemical recognition of their intrinsic molecular properties or by using chemical modification.^22−24^ These remarkable physicochemical and functional features allow hydrogels to be used in biosensing applications.^18,25,26^ So far, a variety of hydrogel-based biosensors have been developed and successfully applied in the selective and sensitive detection of biomarkers or physiological molecules through efficient electrical and optical transduction pathways. Versatile biosensors hold promise for cancer detection due to several advantages. They can be relatively easy to fabricate, offering high sensitivity and a wide range of detection capabilities. Additionally, research suggests that they might be suitable for long-term applications due to their potential robustness, reliability, and reusability.^27^ Specific applications such as the detection of diabetes-related glucose and lactate, cardiovascular disease-related triglyceride, and cancer-related microRNAs and protein, demonstrate their great potential as diagnostic toolsets for disease diagnosis and health monitoring.^28−30^ However, challenges remain before widespread clinical use. Ensuring the longevity, proper storage, and adaptability of these biosensors for rapid and quantitative analysis is crucial. These hurdles suggest further development is needed before they become integrated into commercial health management systems.

In this review, we focus on the development and applications of different hydrogel-based biosensors for cancer detection. We explain the fabrication process of the diverse hydrogel-based biosensors. We highlight the roles of hydrogel-based biosensors in cancer-associated monitoring. We also discuss the potential challenges and limitations in the fabrication and application of these hydrogel-based biosensing platforms. This review provides a focused and up-to-date analysis of advancements in hydrogel-based biosensors for cancer detection (2020–2024), offering valuable insights for researchers in this rapidly evolving field. Particularly, this review bridges a gap by focusing exclusively on the synergy between hydrogels and biosensing for cancer detection within the recent 2020–2024 time frame.

## 2 Hydrogel: A Base Material for Biosensor

**Figure 1:**
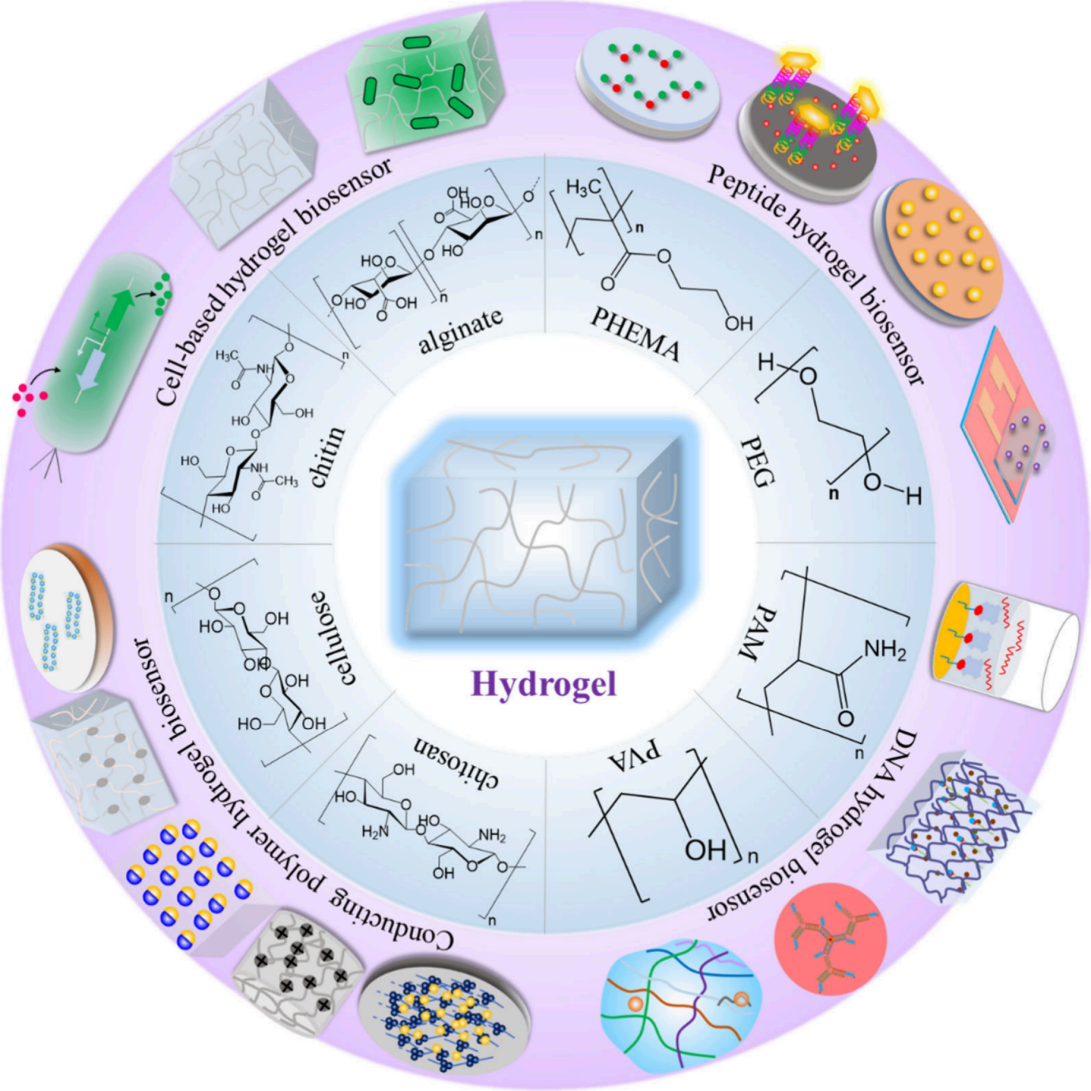
Representation of typical hydrogel-based biosensors.

Hydrogels are three-dimensional networks of polymer chains that can hold a significant amount of water within their structure, forming soft and wet materials. Hydrogels are functional biomaterials that have been in high demand for synthesis, production, and applications in the past few decades. Hydrogels are generally made from synthetic polymers such as poly(2-hydroxyethyl methacrylate) (PHEMA), polyethylene glycol (PEG), polyacrylamide (PAM), and poly(vinyl alcohol) (PVA), or natural polymers such as alginate, chitin, cellulose, and chitosan.^26^ Hydrogels can be produced through physically or chemically cross-linked methods. In physically cross-linked hydrogel, the polymeric network is formed by molecular chain entanglements or some cross-linking interactions such as hydrophobic/hydrophilic interactions, hydrogen bonds, ionic/electrostatic interactions, and crystallization formation.^31^ Chemically cross-linked hydrogels are typically fabricated using reactive functional groups, graft copolymerization, and enzymatic methods through the formation of covalent bonds, which specifically involve photoinduced cross-link, small molecule cross-link, and enzymatically induced cross-link.^32,33^ As functional biomaterials, many hydrogels have been utilized in a vast range of biotechnology, especially as sensory systems, because of their inherent softness, tunable mechanical strength, porous structure with high surface area, ease of functionalization, and good biocompatibility mimicking tissue, making them ideal for sensor development. The synthetic variability enables the hydrogel to have tuning properties to respond to different external stimuli. Upon selective binding of charged biomolecules (e.g., target biomarkers) to the probes immobilized on or within the hydrogel matrix, a measurable change in surface potential is induced. Accordingly, hydrogel-based sensing systems generate direct (current, color, or fluorescence) and/or indirect (UV absorption) read-outs as signal responses.^27^ During some detection processes, the signal can be further amplified, processed, and then displayed.^34^ Examples of commonly used biosensors are electrochemical biosensors,^35,36^ optical-based biosensors,^37,38^ and immunosensors.^39^ By integrating biomolecules or living bacteria into hydrogels, it offers new insights into the design and development of various hydrogel-based biosensors, as summarized in Figure 1.

Hydrogels’ three-dimensional (3D) structure offers significant advantages for biosensing and bioanalytical systems.^40^ While both hydrogels and some plastics can be engineered with similar reactive groups for biomolecule attachment, hydrogels offer several key advantages due to their unique properties. Here’s why hydrogels are a superior choice for biosensing applications:

(1) Mimicking the Cellular Environment: Hydrogels are highly similar to the natural environment of cells and biomolecules. Their high water content (often exceeding 90%) creates a more biocompatible platform compared to a rigid plastic surface. This allows biomolecules to maintain their activity and function more effectively, leading to more accurate and reliable biosensing;
(2) Tailored Properties: Unlike a plain plastic surface, hydrogels offer much greater control over surface properties. By adjusting the composition, pore size, and cross-linking density, hydrogels can be specifically designed to optimize binding with targeted molecules. This leads to highly specific and sensitive biosensors that minimize unwanted interactions;
(3) Signal Amplification: Certain hydrogels can physically change (swell or shrink) in response to biomolecule binding. This change can amplify the signal generated by the interaction, resulting in more sensitive detection.

**Figure 2:**
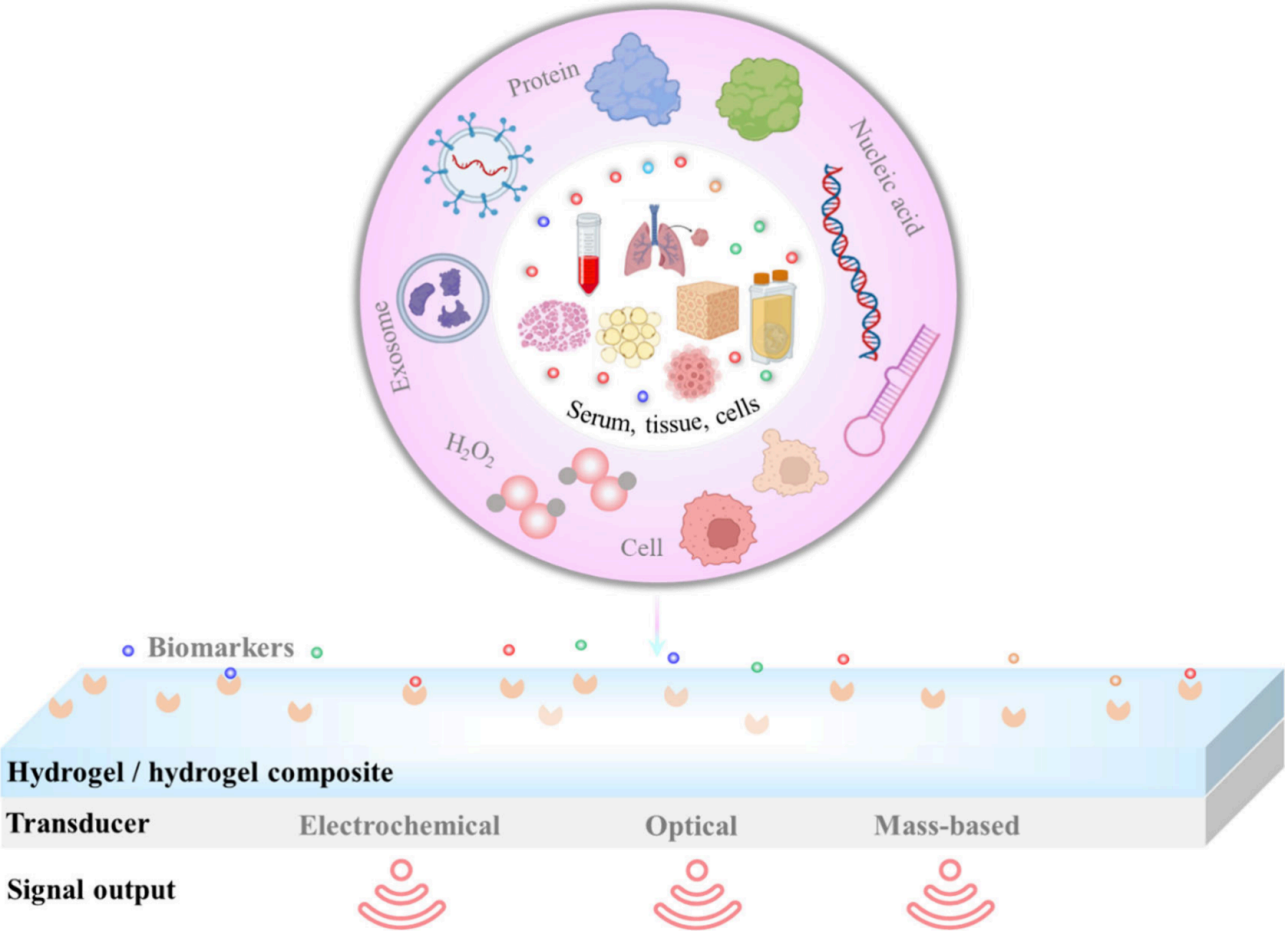
Representation of a hydrogel-based biosensor for cancer biomarkers detection. The human samples (e.g., serum, tissue fluid, cells) are collected and the analytes containing the target biomolecules are extracted. The biomarkers will interact with the bioprobes immobilized on the hydrogel matrix. For signal detection, the affinity interaction will be converted into a measurable signal, such as an electrochemical, optical, or mass-based signal.

Hydrogels can potentially detect very low levels of cancer biomarkers present in the early stages of the disease. Through deploying specific hydrogel, highly selective detection of particular cancer-related biomarkers in real human samples (such as serum, tissue fluid, and cells) can be achieved, which provides a sensitive and noninvasive method for early cancer diagnosis and contributes to the elevated success rate of treatment (Figure 2).

Hydrogel-based biosensors utilize appropriate transducers to convert the biorecognition event into a measurable signal. This more natural environment enhances biomolecule stability and functionality compared to traditional methods.^19^ Several key parameters, including sensitivity, specificity, response time, detection range, minimization of nonspecific binding, and reproducibility, are crucial for evaluating biosensor performance.^41^ These parameters are influenced by the hydrogel’s chemical structure and the physical properties, and the nature and density of cross-links within its network. While hydrogel-based platforms offer numerous advantages, some materials exhibit limitations such as nonspecificity and low conductivity, hindering target recognition and signal transduction.^28^ The advantages and disadvantages of the hydrogel and its comparison with other diagnostic platforms (e.g., microfluidics, porous materials, paper diagnostics, nanopillars) are shown in Table 1. These limitations can be overcome by combining hydrogels with functional materials like metal (e.g., Ag, Au, Pd), or carbon nanomaterials (e.g., carbon dots, carbonized polymer dots, carbon quantum dots) to create hydrogel-based composites.^42^ These composites offer unique performances and diverse functionalities, allowing for the integration of multiple biosensing components into a single system for efficient molecular detection.

**Table 1:** Advantages and Disadvantages of Different Diagnostic Platforms

diagnostic platforms	advantages	disadvantages
Hydrogels	(1) Biomimetic environment: Mimics the natural cellular environment, allowing for compatibility with living cells and enzymes.	(1) Limited shelf life: May degrade over time, requiring careful storage and handling.
	(2) High water content: Facilitates diffusion of biomolecules and enhances sensitivity.	(2) Diffusion limitations: Larger molecules may have slower diffusion rates within the hydrogel.
	(3) Tunable properties: Can be designed with specific properties (e.g., stiffness, porosity) for targeted applications.	(3) Limited functional properties: Hydrogels tend to be nonconductive and nonspecific without metals or other supporting materials.
	(4) Encapsulation capabilities: Can immobilize enzymes, antibodies, living cells, and other biorecognition elements for specific detection.	(4) Potential for nonspecific binding: This can lead to false positives if not carefully optimized.
Microfluidics	(1) High throughput: Can analyze multiple samples simultaneously and rapidly.	(1) Complexity: Requires specialized equipment and technical expertise for fabrication and operation.
	(2) Precise manipulation: Enables precise control over fluid flow and reaction conditions.	(2) Cost: Can be expensive to fabricate and utilize.
	(3) Miniaturization: Can be miniaturized for point-of-care applications.	(3) Limited biocompatibility: Some materials used in microfluidics might not be compatible with biological samples.
Porous materials	(1) High surface area: Provides ample space for capturing biomolecules and enhancing detection sensitivity.	(1) Limited control over biomolecule orientation: Can affect binding efficiency and specificity.
	(2) Tailorable pore size: Can be designed with pores of specific sizes to selectively capture target molecules.	(2) Potential for clogging: Pores can become clogged by biological debris, hindering performance.
	(3) Cost-effective: Can be relatively inexpensive to fabricate.	(3) Limited reusability: Depending on the material, may not be easily reusable.
Paper diagnostics (Lateral flow assays)	(1) Simplicity: Easy to use, requiring minimal training or equipment.	(1) Limited sensitivity: May not be as sensitive as other techniques for detecting low levels of analytes.
	(2) Portability: Lightweight and portable, suitable for resource-limited settings.	(2) Limited multiplexing: Typically limited to detecting one or a few targets simultaneously.
	(3) Low cost: Relatively inexpensive to manufacture and utilize.	(3) Short shelf life: Paper-based assays may degrade over time, limiting their storage life.
Nanopillars	(1) Ultrahigh surface area: Provides an extremely large surface area for capturing biomolecules, enhancing sensitivity.	(1) Complexity: Requires advanced fabrication techniques and specialized equipment.
	(2) Label-free detection: This can potentially enable detection without the need for labels or markers.	(2) Cost: Can be expensive to fabricate and utilize.
	(3) Real-time monitoring: This may allow for real-time monitoring of biomolecule interactions.	(3) Limited development: Still in the early stages of development, requiring further research for practical applications.

## 3 Hydrogel-Based Biosensor for Cancer Detection

Leveraging the diverse building blocks employed in hydrogel materials (e.g., peptide, DNA, conducting polymer), researchers have developed a variety of hydrogel-based biosensors with outstanding potential for cancer detection, as evidenced by their increasing adoption in biosensing and diagnostic tools (Table 2).

**Table 2:** Comparison of Performance Metrics of Various Hydrogel-Based Biosensors^a^

biosensors	target	response time	sensitivity	detection range	ref.
Peptide hydrogel biosensors	HER2	60 min	45 pg/mL	0.1 ng/mL to 1.0 μg/mL	(43)
	MMP-7	70 min	24.34 fg/mL	0.1 pg/mL to 100 ng/mL	(44)
	PSA	80 min	5.6 pg/mL	0.1 ng/mL to 100 ng/mL	(45)
	Tyrosinase	24 h	1.9 fM	10 fM to 1 nM	(46)
DNA hydrogel biosensors	Exosomes	N/D	22 μL^–1^	0 to 100 nmol/L	(47)
	miR-17	20 min	182 aM	10 aM to 10 nM	(48)
	ATP	60 min	500 cells/μL	0 to 5000 cells/μL	(49)
Conducting polymer hydrogel biosensors	CEA	60 min	0.06 fg/mL	1 fg/mL to 200 ng/mL	(50)
	DA	N/D	2.0 pmol/mL	0.01 to 100 nmol/mL	(51)
	H_2_O_2_	N/D	9.6 μM	50 to 3000 μM	(52)

a human epidermal growth factor receptor 2 (HER2), metal matrix protease-7 (MMP-7), prostate specific antigen (PSA), carcinoembryonic antigen (CEA), dopamine (DA), not determined (N/D).

### 3.1 Peptide Hydrogel Biosensor

Peptide hydrogels are natural hydrogels that are made of peptides or polypeptides, which are chains of amino acids linked by peptide bonds (−CONH−). These hydrogels can be formed by physical or chemical cross-linking of the peptide chains.^53^ Peptide hydrogels are natural self-assembled materials that have many advantages, such as being designable, biodegradable, nontoxic, inexpensive, and hypoimmunogenic.^54^ These features make them suitable for various applications in materials science, biomedicine, tissue engineering, and biosensors.^55^

**Figure 3:**
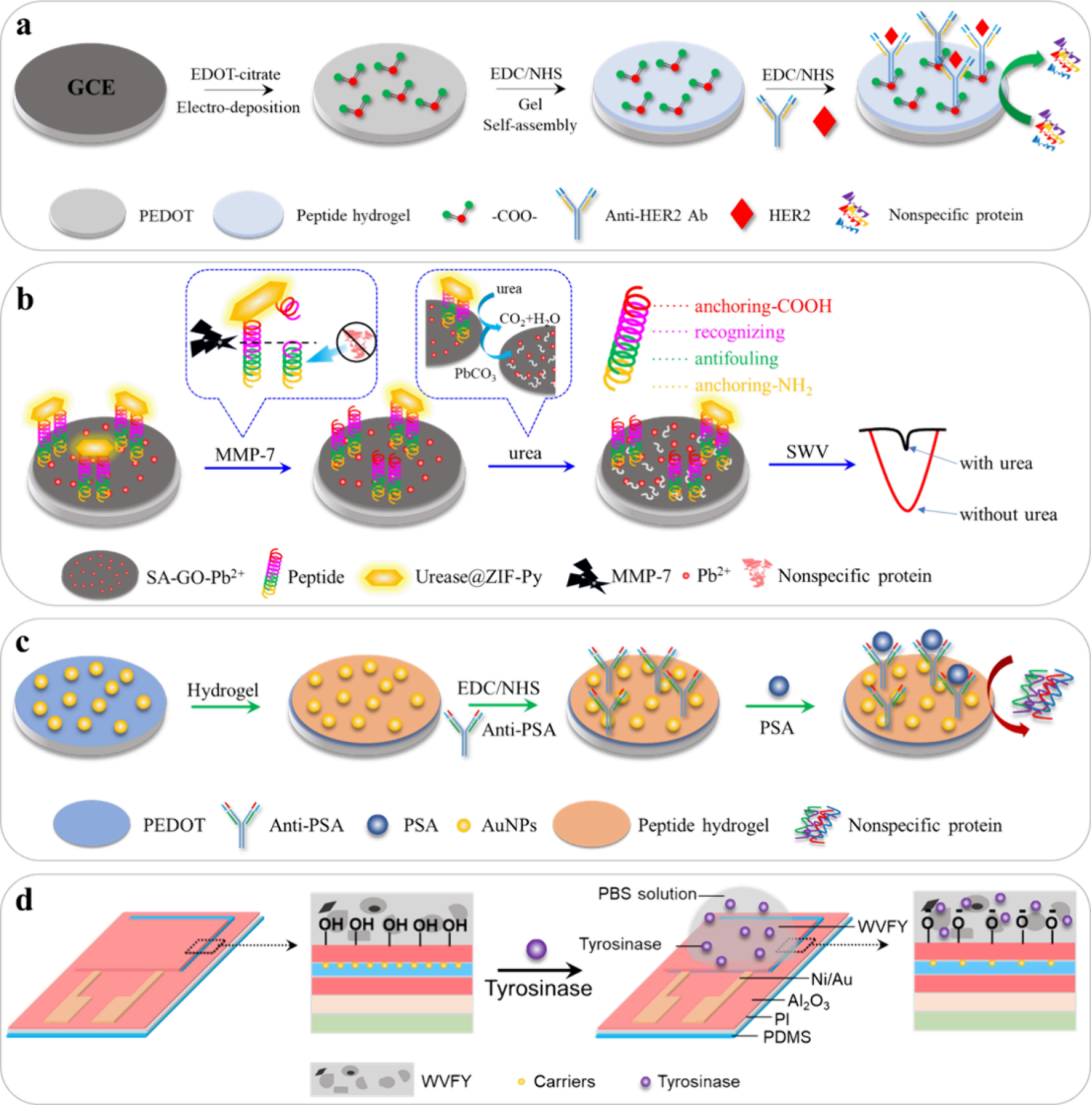
Illustrations of the preparation process of developed peptide hydrogel-based biosensors. (a) PEDOT/peptide hydrogel-based HER2 biosensor. (Reprinted and adapted with permission from ref (43). Copyright 2021 American Chemical Society.) (b) Electrochemical biosensor developed based on a strategy of combining a multifunctional peptide with urease@zeolite imidazole frameworks. (Reprinted and adapted with permission from ref (44). Copyright 2022 Elsevier.) (c) The fabrication process of the PSA electrochemical biosensor based on the antifouling zwitterionic peptide hydrogel. (Reproduced with permission from ref (45). Copyright 2023 Elsevier.) (d) Fabrication process of Al_2_O_3_/In_2_O_3_ FET-based biosensor. (Reprinted and adapted with permission from ref (46). Copyright 2022 Elsevier.)

Among them, peptide hydrogel biosensors have attracted increasing attention in recent years due to their high responsiveness to external stimuli. During the fabrication of highly sensitive biosensors, peptide hydrogels are usually applied in combination with certain sensing molecules, such as label molecules, conductive materials, DNA strands, and enzymes, which also can prevent nonspecific binding on the gel surface.^56^ For instance, Wang et al. developed an antifouling sensing interface using the poly(3,4-ethylenedioxythiophene)-based peptide (designed as Phe-Glu-Lys-Phe) hydrogel for the detection of a breast cancer biomarker (human epidermal growth factor receptor 2)^43^ (Figure 3a). The peptide hydrogels encapsulating horseradish peroxidase (HRP) were formed by diluting the peptide stock solutions with an aqueous HRP solution in 96-well plates. The PEDOT/gel interface was fabricated on the bare GCE surfaces using a potentiostatic method, followed by immobilization of the HER2 antibody to prepare a stable GCE/PEDOT/Gel/Ab biosensor. The peptide hydrogel with a dense fibrous network contributed a hydration layer to resist the nonspecific adsorption and a biocompatible microenvironment for the HRP to retain high bioactivity. The biosensors’ overall hydrophilic surfaces minimized nonspecific adsorption. Specific binding of the HER2 target to the immobilized antibody on the interface resulted in a decrease in the current signal. This decrease is attributed to the formation of a dielectric antibody–antigen complex on the sensing surface, which hinders electron transfer. The observed decrease in differential pulse voltammetry (DPV) current signal with increasing HER2 concentration enables the constructed hydrogel-based biosensor to perform quantitative analysis of HER2. As a result, the highly sensitive and selective biosensor displayed a low LOD of 45 pg/mL and a wide linear response range from 0.1 ng/mL to 1.0 μg/mL. By combining a multifunctional peptide hydrogel with urease@zeolite imidazole frameworks, Zhang et al. constructed an accurate and low-fouling sensing platform to accurately detect tumor biomarker matrix metalloproteinase-7 (MMP-7) in the biological samples^44^ (Figure 3b). The ethylenediamine (EDA)/urease@ZIF-Py was synthesized by mixing ZnAc_2_, urease, pyrrole-2-carboxylic acid (0.56 M) and 2-MeIM (0.28 M) at room temperature for 1 h. Next, the SA-GO-Pb^2+^ hydrogel sensing platform was fabricated based on the mixture of 0.04% SA solution, 0.005% GO suspension, and 0.1 M Pb(NO_3_)_2_. Subsequently, an appropriate amount of peptide solution and EDA/urease@ZIF-Py were placed onto the electrode interface. The lysine (NH_2_–K) and glutamic (E-COOH) residues at both ends of the peptide can connect the carboxyl group of the hydrogel and the amino group of ZIF-Py through amide bonds. The electrically neural property of the EKEKEK sequence and excellent hydrophilicity of the pep/SA-GO-Pb^2+^/GCE surface contributed to the enhanced antifouling performance of the sensing interface. When MMP-7 and urea were incubated on the sensing interface sequentially, the current signal first rose sharply, which was attributed to the specific hydrolysis of the peptides on the sensing interface by MMP-7; then, due to the precipitation of PbCO_3_ caused by urea incubation, the current signal began to decrease. The biosensor exhibited high sensitivity and significant antifouling ability, with a low LOD of 24.34 fg/mL and a broad linear range from 0.1 pg/mL to 100 ng/mL. Similarly, Du et al. fabricated an antifouling zwitterionic peptide-based (CFEFKFC) hydrogel biosensor to determine a cancer biomarker, namely prostate-specific antigen (PSA), in complex human serum samples^45^ (Figure 3c). In brief, the PEDOT/AuNPs/hydrogel-based antifouling interface was constructed by placing 5.0 μL CFEFKFC-based peptide hydrogel onto the PEDOT/AuNPs modified electrode surface for 12 h in a humid chamber. Followed by the installation of anti-PSA antibodies onto the carboxyl groups on the PEDOT/AuNPs/hydrogel electrode via amide bonds, the PEDOT/AuNPs/hydrogel/Ab-based biosensor was fabricated. The strong affinity between the hydrogel and water molecules responsible for the formation of a hydrated shell on the hydrogel surface, and the excellent hydrophilicity of CFEFKFC-based hydrogel facilitate the great antiprotein adsorption capacity of the biosensor. The capture of the PSA target by the anti-PSA antibody immobilized on the electrode caused a decrease in the DPV peaks. This was because the antigen–antibody conjugate hindered the electron transfer at the sensing interface, resulting in a decrease in the electrochemical signal, thereby achieving quantitative PSA detection by monitoring the changes in the DPV current signal. The developed biosensor showed excellent antifouling property, with a low LOD down to 5.6 pg/mL and a satisfactory detection range from 0.1 ng/mL to 100 ng/mL. In the study of Ren et al.,^46^ a highly sensitive biosensor was constructed by self-assembling nanostructured tetrapeptide (WVFY) on metal-oxide transistors for the sensing of tyrosinase, a melanoma biomarker (Figure 3d). The fabrication process involved sequential deposition of a thin Al_2_O_3_ buffer layer and an In_2_O_3_ channel layer onto a polyimide (PI) film. Interdigitated Ni/Au electrodes were then embedded in the layered PI film to create Al_2_O_3_/In_2_O_3_ bio-FET arrays. Subsequently, these arrays were then transferred to a polydimethylsiloxane (PDMS) substrate and functionalized with WVFY peptides through self-assembly. The sensing mechanism relies on the specific interaction between tyrosinase and the surface-modified bio-FETs. In the presence of tyrosinase, the phenolic hydroxyl group of the WVFY peptide undergoes oxidation to catechol and further to benzoquinone, with concomitant consumption of protons, leading to electrostatic repulsion and a decrease in channel conductance. This change in conductance is manifested as a shift in the threshold voltage (*V*_th_) shifts of bio-FETs, which can be measured potentiometrically. The wearable peptide-modified biosensor film was able to detect tyrosinase with high sensitivity, displaying an ultralow LOD of 1.9 fM and an optimal detection range from 10 fM to 1 nM. Preparing highly conductive and transparent soft electronic devices and wearable biosensors is considered a huge challenge. Therefore, Jing et al.^57^ developed a method to produce conductive nanofibrils in dipeptide hydrogel networks by incorporating hydrophilic and conductive nanoparticles made of polydopamine (PDA) and polypyrrole (PPy). These nanofibrils had high transmittance and excellent conductivity, making them suitable for body-based sensing platforms. Updating these platforms can enable more advanced biomolecular analysis and physiological monitoring.

When new peptide molecules and peptide assemblies combined with other advanced materials are encapsulated into a hydrogel network, they can become novel biosensors for cancer detection. During the construction of versatile biosensors, peptides are designed to have specific sequences, substrate recognition, and analyte affinity. However, there are still many challenges to overcome. One of them is the molecular recognition mechanism, which is a key limitation of the current peptide-based biosensing platforms.^58^ As new cancer biomarkers continue to be discovered, accordingly, there is an increasing need for new peptide components and the construction of new peptide sensing principles. Another one is the detection of multiple cancer biomarkers within a single biosensor, which is highly desirable. On the basis of the theoretical knowledge of bioinformatics and structural biology, multifunctional peptide hydrogels with excellent sensing ability may be generated. A third one is that most current peptide-based hydrogel biosensors have poor performance in real human samples (e.g., blood, serum, urine, and interstitial fluid).^59^ The complex components cause significant background noise leading to inaccurate molecular recognition. The nonspecific molecular adsorption in complicated systems also limits the clinical use of these biosensors. The development of the antifouling systems of the biosensor can potentially overcome this issue, but most of them are still in their early stages.

In general, chemically cross-linked peptide hydrogels offer greater stability, but they might introduce potentially cytotoxic chemicals that could affect the sensitivity and stability of living biosensors.^60,61^ Conversely, physically cross-linked peptide hydrogels, while potentially less stable structurally, may be less likely to compromise biosensor function in the long term.^62,63^ Therefore, further research is necessary to optimize hydrogels for enhanced biosensor stability.

Moreover, the synthesis and fabrication process of some peptide-based hydrogel combined materials are complex and costly, which increases the production cost of these biosensors.^64^ A simplified synthesis approach and factory design are needed. It is hoped to further reduce production costs by multidisciplinary collaboration of material science, polymer chemistry, and biochemistry. Despite these challenges, biosensors have created a new opportunity for research and industry, and we expect that peptide-based biosensing platforms will be clinically applied in the near future.

### 3.2 DNA Hydrogel Biosensor

DNA hydrogels consist of highly cross-linked polymeric networks formed by cross-linking and hybridization of DNA molecules.^65^ DNA-based hydrogels have many advantages, such as mechanical rigidity, biocompatibility, biodegradability, high physicochemical stability, and programmability.^66−68^ These properties make them suitable for various applications, especially biosensing.^66^ DNA hydrogels can be designed to be specific, sensitive, portable, and low-cost biosensors. In the past few decades, researchers have used DNA hydrogel biosensors for various purposes, such as biosensing, drug development, molecular diagnostics, and cancer detection.^69^

**Figure 4:**
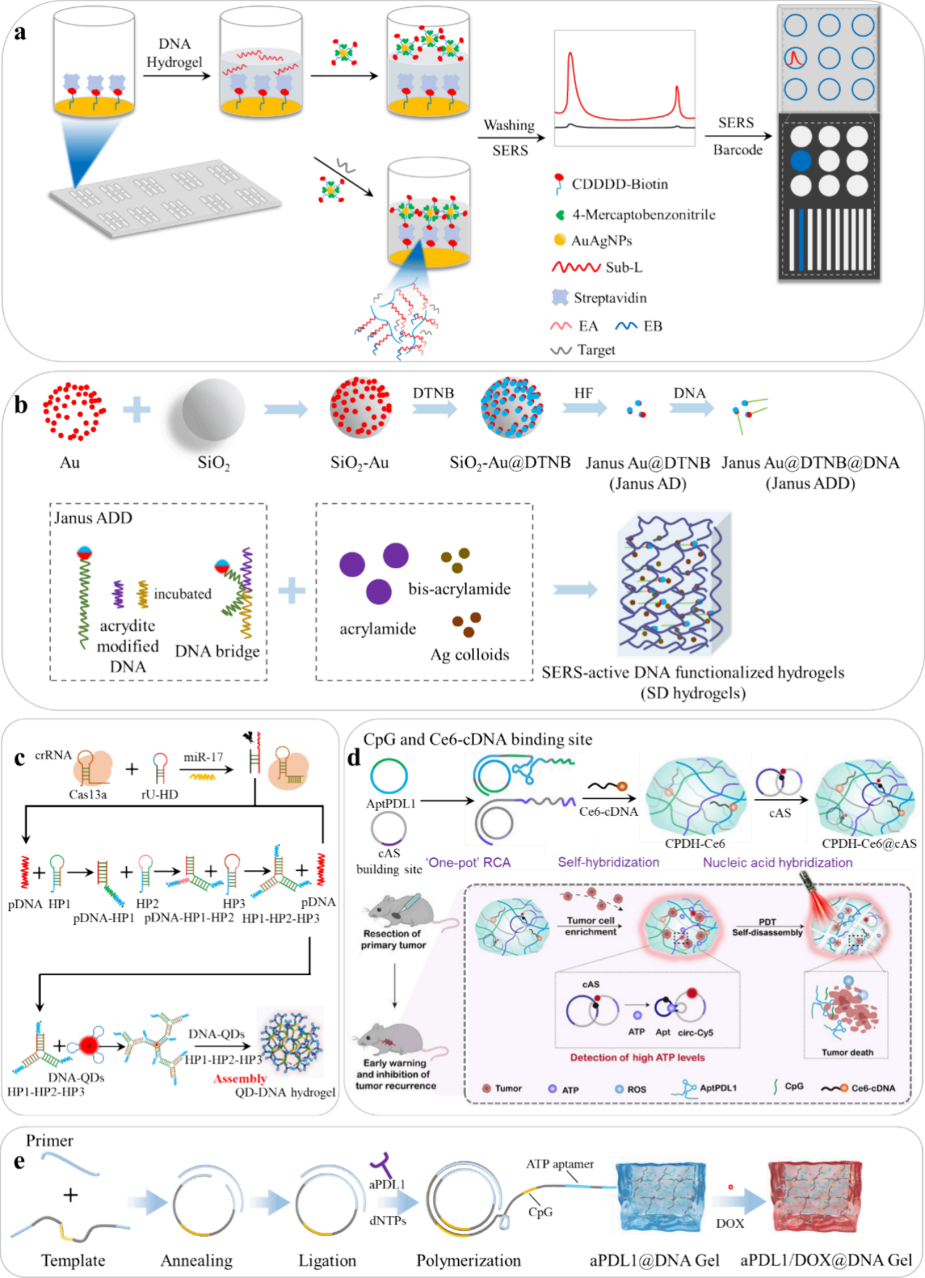
Descriptions of DNA hydrogel biosensors fabrication process. (a) Production and application of the target miRNA-responsive DNA hydrogel-based SERS biosensor array for multiple miRNAs detection in one sample. (Reprinted and adapted with permission from ref (70). Copyright 2020 American Chemical Society.) (b) Illustrations of SD hydrogel preparation. (Reproduced and adapted with permission from ref (47). Copyright 2023, Acta Optica Sinica) (c) Illustration of the design principle of the Cas-TMSD-QDH used for miR-17 detection. (Reprinted and adapted with permission from ref (48). Copyright 2023 Elsevier.) (d) Construction of a hydrogel with integrated diagnostic and immunotherapeutic capabilities. (Reproduced and adapted with permission from ref (49). Copyright 2023, Springer Nature.) (e) Fabrication process of aPDL1/DOX@DNA hydrogel. (Reprinted and adapted with permission from ref (71). Copyright 2023, Wiley).

For example, Si et al. fabricated a new surface-enhanced Raman scattering (SERS) sensor array based on a target microRNA (miRNA)-responsive DNA hydrogel with nine sensor units, which can detect multiple cancer-related miRNAs in one sample^70^ (Figure 4a). The fabrication of the biosensor includes construction of the streptavidin-modified sensor surface and production of the target miRNA-responsive DNA hydrogel. Initially, the SERS tag could not pass through the hydrogel to bind to the streptavidin (SA)-modified sensing surface because the formed DNA hydrogel blocked the SA-modified sensing unit, thereby no obvious Raman signal could be observed. When the target miRNA was introduced, the DNA hydrogel of the corresponding sensing unit disintegrated, and the SERS tag was able to pass through the hydrogel and be captured on the SA-modified detection surface, thereby generating a strong Raman signal, realizing the detection of the target miRNA. The assay was validated by detecting several miRNAs such as miR-21, miR-221, miR-224, etc., which were associated with breast cancer, pancreatic cancer, liver cancer, and many other types of cancers in both clean buffer and serum samples. Yang et al. developed surface-enhanced Raman spectroscopy (SERS)-active DNA functionalized hydrogels (SD hydrogels) for the detection of tumor-derived exosomes^47^ (Figure 4b). The detection ability of SD hydrogels was proved by the complementary aptamers at different concentrations ranging from 0 to 100 nmol/L in a phosphate-buffered saline (PBS) solution. The SERS intensity of DTNB in SD hydrogels distinctly decreased with the increased concentration of complementary aptamers, indicating that SD hydrogels were suitable for biological detection. The LOD of the present DNA hydrogel biosensor was found to be approximately 22 μL^–1^. Yang et al. designed a new CRISPR-Cas-catalyzed formation of quantum dot-DNA (QD-DNA) hydrogel-based detection system (Figure 4c). On the basis of the seed-mediated growth method, the CdTe/CdS QDs was fabricated by growing the CdS shell on the surface of the CdTe core. Then the DNA-CdTe/CdS QDs (DNA-QDs) were prepared using phosphorothioate-modified DNA (ps-po DNA) to direct the growth of the CdS shell. The Cas-TMSD-QDH assay utilized toehold-mediated strand displacement (TMSD) amplification catalyzed by CRISPR-Cas13a trans-cleavage-released products and detected target miRNAs through self-assembled QD-DNA hydrogels in which QDs were efficiently quenched. It was further applied for the highly sensitive detection of miR-17 levels in several cell lines (such as ZR-75–30, MCF-10A, and MCF-7), with the lowest LOD being 182 aM.^48^ Wang et al. developed a special DNA hydrogel with an immunomodulatory function that can be used for early monitoring and inhibition of postoperative tumor recurrence^49^ (Figure 4d). To prepare Ce6 and cAS-loaded hydrogels (CPDH-Ce6@cAS), CPDH-Ce6 was initially achieved by one-pot rolling circle amplification of two partially complementary circular DNA templates and Ce6-cDNA. For the preparation of the cyclic ATP sensor, circular DNA (C1) was first formed via template-based ligation using linear DNA (L1) and primers. The circular ATP sensor (cAS) was prepared by mixing C1 (2 μM) and 5′-phosphorylated linear DNA 2 (L2:10 μM), followed by incubation of cAS and CPDH-Ce6 for 1 h. In principle, some PDL1 aptamers in the DNA hydrogel could capture and enrich relapsed tumor cells in situ and increased local ATP concentration to yield a timely signal of warning. In a mouse model in which tumor cells were injected into the surgical site to simulate tumor recurrence, the hydrogel system was found to be able to detect tumor recurrence in a timely manner by enriching recurrent tumor cells to increase local ATP concentration. Similarly, Fan et al. reported a new injectable stimuli-responsive immunomodulatory depot by programming a supersoft DNA hydrogel adjuvant^71^ (Figure 4e). The DNA template was carefully designed with complementary sequences for the ATP aptamer and CpG ODN. To achieve the polymerization of rolling circle amplification, Phi29 DNA polymerase was utilized to extend DNA primers on enzymatically circularized DNA templates and noncovalently weave them into a hydrogel network. The developed hydrogel system encoded with an ATP aptamer can be injected intratumorally into a gel formulation and then stimulates different release kinetics of coencapsulated therapeutics under significant molecular conformational changes.

Despite the rapid development of DNA hydrogel-based biosensors, several challenges that limit its practical applications need to be addressed. First, making DNA hydrogel is complex, time-consuming, and costly. This limits their scalability for clinical use, such as cancer diagnosis and treatment. Second, the DNA molecules in the hydrogels are prone to enzymatic degradation. This reduces their stability and effectiveness in the body.^68^ When applied in biomedical situations, their great stability in vivo and their ability to remain intact and effective during blood circulation are required. The appropriate diffusion and release time are required. Third, DNA nanomaterials are thought to trigger an immune response, potentially resulting in some adverse effects.^72^ Fourth, the DNA hydrogel biosensors have low sensitivity, which means that they cannot detect small changes in the target molecules or the cancer cell microenvironment. And the detection object is relatively single, which needs to be optimized for real detection settings. Fifth, the DNA hydrogel biosensor has been used to detect only a small number of tumors. It is necessary to broaden the detection of other tumors. To overcome these challenges and exploit the opportunities, more research and development are needed. One possible solution is to design intelligent DNA hydrogels that can respond more efficiently to biological molecules and improve the diffusion time of the cargo.^73^ Another possible solution is to understand the mechanisms of DNA hydrogel release and metabolism in the body. This will help to optimize the biosensor and therapeutic performance. The programmability of DNA nanostructures may enable the development of smart therapeutic systems and stimulus-responsive DNA hydrogel biosensor platforms. These systems and platforms will be able to sense changes in the cancer cell microenvironment and release distinct therapeutics on demand.

### 3.3 Conducting Polymer Hydrogel Biosensor

Synthetic polymers are frequently employed as matrices for hydrogel production because they offer a stable, chemically stable, electrically conductive, and biosensing-friendly platform. Synthetic polymers such as polyacrylamide (PAAm),^74,75^ polypyrrole,^76^ poly(acrylic acid) (PAAc),^77,78^ polyaniline,^79^ poly(vinyl alcohol) (PVA),^80^ and poly-2-hydroxyethyl methacrylate (PHEMA)^81^ are some of the most common and widely used synthetic materials. They have low density and easy synthesis, which make them suitable for forming hydrogel networks and conducting polymer hydrogels (CPHs). CPHs have emerged as an amazing class of novel materials that combine the benefits of both conductive polymers and 3D hydrogels. They have remarkable electrical and mechanical properties.^82^ Therefore, CPHs are widely used as biomaterials for various applications, such as soft tissue, drug delivery, tissue engineering, bioimaging, and wearable biosensors.^83^

**Figure 5:**
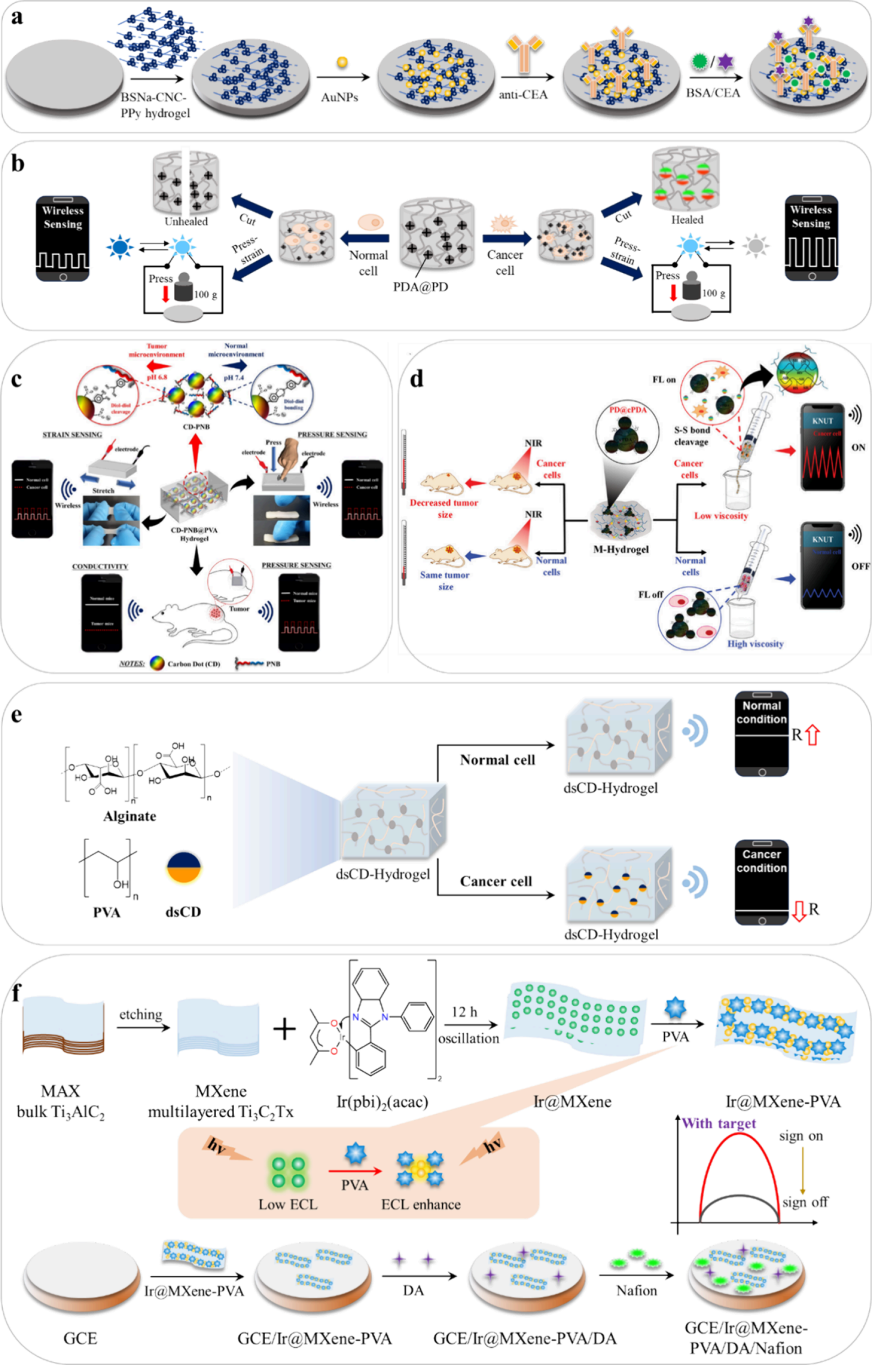
Illustration of design processes of the developed conducting polymer hydrogel biosensors. (a) Illustration of the fabricated immunosensor for CEA detection. (Reprinted and adapted with permission from ref (50). Copyright 2021, The Royal Society of Chemistry.) (b) Illustration of strain–stress hydrogel-based biosensor for cancer detection with real-time monitoring function using a smartphone. (Reprinted and adapted with permission from ref (85). Copyright 2021 Elsevier.) (c) Representation of the CD-PNB@PVA hydrogel for strain-pressure-based tumor detection. (Adapted with permission from ref (86). Copyright 2021 Elsevier.) (d) Illustration of the cancer-selective nature of M-Hydrogel and its application. (Adapted with permission from ref (88). Copyright 2023, Wiley) (e) Model of the dsCD-Hydrogel with the change in conductivity for cancer detection. (Reproduced and adapted with permission from ref (89). Copyright 2023 Elsevier) (f) A diagram of the preparation process of Ir@MXene-PVA and building of the ECL sensing platform. (Reprinted and adapted with permission from ref (51). Copyright 2024 Elsevier.)

Biosensors are one of the main fields that benefit from CPHs.^11^ Many studies have reported on the use of conducting polymers from CPHs in biosensors. CPHs can be used as standalone devices or as implantable sensors.^84^ CPHs-based biosensors can detect different types of molecular biomarkers, using various analytical and technological methods. For instance, Huang et al. developed a novel label-free electrochemical immunosensor using a nanostructured CPH combined with gold nanoparticles (AuNPs) for delicate measurement of carcinoembryonic antigen (CEA), a biomarker of many cancers (Figure 5a). This 3D nanohydrogel (BSNa-CNC-PPy) was fabricated through synthesis of polypyrrole (PPy) using cellulose nanocrystalline (CNC) and sodium benzenesulfonate (BSNa) as dopants.^50^ The fabrication process involved mixing 1.12 M ammonium persulfate, 4 mg/mL CNC, 1.33 M pyrrole (Py), and BSNa to prepare the BSNa-CNC-PPy gel. This solution was then dropped onto the glassy carbon electrode (GCE) and allowed to polymerize at 4 °C for 25 min. Subsequently, gold nanoparticles (AuNPs) and 200 μg/mL anti-CEA antibodies were sequentially deposited on the gel/GCE to create the final immunosensor. CNC played a dual role as a dopant and gelator in this system. Through hydrogen bonding and electrostatic interactions with PPy chains, CNC facilitated the polymerization of the PPy-based nanonetwork. This resulted in a biosensor with enhanced conductivity, a larger specific surface area, and a more hydrophilic interface. BSNa, acting as a dopant, improved the conductivity and stability of the substrate by promoting interchain charge transport within the PPy network. The sensing mechanism relies on the specific interaction between CEA and the immobilized anti-CEA antibody. Upon incubation with CEA, the formation of an antigen–antibody complex on the AuNPs/BSNa-CNC-PPy gel/GCE electrode hinders electron transfer, leading to a decrease in the current signal. This change in current response is monitored using square wave voltammetry (SWV) and electrochemical impedance spectroscopy (EIS) techniques. Collectively, the CNC-PPy gel/glassy carbon electrode (GCE) exhibited high conductivity, strong hydrophilicity, biocompatibility, nanostructure, and excellent film-forming properties. The developed CEA-detecting biosensor showed an ultralow LOD (0.06 fg/mL) and a broad detection range (1 fg/mL to 200 ng/mL). Robby et al. designed a polydopamine-loaded glutathione-responsive polymer dot (PDA@PD) hydrogel-based electronic-skin sensor with high selectivity toward CD44 receptor for the detection of cancer cells (such as MDA-MB-231, KB and HeLa cancer cell lines)^85^ (Figure 5b). The disulfide-cross-linked PD was synthesized via hydrothermal carbonization at 180 °C for 8 h, followed by loading of PDA nanoparticle into PD. The resulting PDA@PD nanoparticles were incorporated into the PAAm matrix to obtain PDA@PD–PAAm hydrogel through in situ radical polymerization at room temperature for 6 h. In the PDA@PD complex, PDA nanoparticles were internalized in the hydrophobic core of disulfide-cross-linked PD via hydrophobic interactions or π–π stacking, facilitating electron transfer, thus increasing electrical conductivity. In the presence of an excessive amount of glutathione in cancer cells, the disulfide bond in PD was cleaved and allowed the release of PDA which triggered an electroconductivity change. These hydrogel-based sensors therefore produced significantly different conductivity and electronic strain-pressure responses to the conditions in a tumor microenvironment. In a similar study conducted by Robby et al.,^86^ a wireless strain-pressure hydrogel sensor was designed based on the pH-responsive nanoparticles (CD-PNB) for cancer detection (Figure 5c). The CD-PNB was synthesized by diol–diol cross-linking between catechol groups in semiconducting carbon dots (CDs) and boronic acid groups in a nonconductive polymer (PNB). Na^+^ ions were added into CD-PNB to increase the electroconductivity through HPC and Na^+^ ionic complex interactions. These were further introduced into PVA matrix using a freezing-thawing method. The CD-PNB@PVA hydrogel biosensor generated different electronic signals when used for detection of cancer cells (HeLa and PC-3), exhibiting higher strain-pressure sensitivity in the comparison to the normal cells (MDCK and CHO-K1). In principle, when strain and pressure were applied to the CD-PNB@PVA hydrogel, the cleavage of the diol–diol bond between the catechol group in the CD and the boronic acid group in PNB resulted in a more acidic condition, different conductivities, and electronic responses due to the unconnected semiconducting CD nanoparticles and nonconductive PNB.

More recently, Lee et al. leveraged thermodynamic partitioning of hydrogel components to create ultrasoft hydrogel microdroplets in which the oil phase is composed of kerosene and polyglycerol polyricinoleate.^87^ To enhance visibility within dense light-scattering tissues, 0.2 μm diameter carboxylate-modified fluorescent polystyrene particles were added to the prepolymer mixture. The stress sensor was then applied in inducible models of breast cancer tumor invasion and was shown to be able to distinguish internal stress patterns caused by cell-matrix interactions at different stages of cancer progression. Roy et al. proposed a redox-responsive mineralized conductive hydrogel-based (termed as M-Hydrogel) cancer-selective self-reporting biosensor.^88^ The GSH-responsive polymer dots (PD) were fabricated by hydrothermal process-assisted carbonization of a disulfide cross-linked polymer (Alg-S-S-Alg). The PD was then loaded onto the carbonized polydopamine (cPDA) via hydrophobic interactions or π–π stacking. The redox-triggered release of cPDA from the disulfide-cross-linked PD@cPDA-loaded hydrogel through the cleavage of disulfide bonds resulted in the activation of the macroporous structure of the self-recognizable M-Hydrogel sensor, thus controlling the conductivity and fluorescence, which was responsible for the self-recognizable sensing ability to GSH-enriched cancer cells (Figure 5d). In mice with HeLa cell xenografts, the use of the wireless biosensing system enabled real-time measurement of the upregulation of pro-apoptotic biomarkers (*P53* and *BAX*) in tumors. Similarly, Jo et al. fabricated a biosensing platform based on the wireless stress and strain sensing response induced by the reactive oxygen species (ROS)-responsive carbon dots present in conductive PVA/Alg [Poly(vinyl alcohol) and sodium alginate] hydrogel for the cancer microenvironment-selective detection^89^ (Figure 5e). The hydrogel was made by introducing dsCD into a PVA-Alg matrix to form a dsCD-embedded double network hydrogel. When the high concentration of ROS was supplied, the diselenide bond was broken and the particle size became smaller, increasing the fluorescence intensity. The resulting particles also had an increased charge carrier density. As a result, differences in conductivity and pressure-strain response were found between normal and cancer cells. In contrast to normal cells, the ROS-responsive hydrogel with high strain-pressure sensitivity produced distinct electronic signals for the detection of cancer cells. Moreover, it also demonstrated a remarkable stress-sensing response in tumor-bearing mice using ex-situ measurements.

Hydrogen peroxide (H_2_O_2_) is a key reactive oxygen species (ROS) that regulates various biological processes and is linked to diseases ranging from obesity to cancer.^90−92^ It also acts as a vital signaling molecule in living organisms and as a promising cancer biomarker.^93,94^ Compared to the normal cells, the cancer cells produced higher levels of H_2_O_2_ in their microenvironment.^95^ Therefore, a number of polymer hydrogel-based biosensors have been designed and developed for H_2_O_2_ detection.^96^ For instance, a sensing system based on Ti_3_C_2_T_*x*_ MXene-stabilized iridium bis(1, 2-dipheny1–1 H-benzimidazole) (acetylacetonate) [Ir(pbi)_2_(acac)] with AIECL in poly(vinyl alcohol) polymer hydrogel was developed for the detection of dopamine (DA) necessary for early diagnosis and treatment of many cancers^51^ (Figure 5f). DA and H_2_O_2_ were able to quench ECL of Ir@MXene-PVA systems. The biosensing system showed a detection range of DA of 0.01–100 nmol/mL and displayed a LOD of 2.0 pmol/mL in human serum. Zhang et al. prepared a 3D network structure polyaniline hydrogel using phytic acid (PA) and hydrochloric acid (HCl) as dopants.^52^ PA has both doping and cross-linking functions, while HCl as a dopant to codope polyaniline has strong ionization ability. The conductive polyaniline introduces a number of hydrophilic groups and forms a cross-linked microscopic network. The hydrogel solution was then applied dropwise to the surface of the pretreated glassy carbon electrode to create (HCl/PA-CPAniH)/GCE. Based on the three-dimensional network skeleton, HCl/PA-CPAniH utilized its good electrical conductivity to conduct charges for the reduction process of H_2_O_2_ and contributed to the rapid transport of H_2_O_2_ and other particles. Ag NPs@PA was added into the hydrogel-based system to produce a new electrochemical biosensor (Ag NPs@PA/(HCl/PA-CPAniH)/GCE) for the detection of H_2_O_2_. The sensor had a good linear relationship, a LOD of 9.6 μM, and a sensitivity of 51 μA/mM/cm^2^. Subba et al. developed a cancer-specific dopamine-conjugated sp^2^-rich carbonized polymer dot (PD)-encapsulated mesoporous MnO_2_ (MnO_2_@PD)-mineralized hydrogel biosensor.^97^ MnO_2_@PD nanoparticles were prepared by mixing 1 mg/mL PD and 5 mg MnO_2_ mesoporous under stirring for 24 h, which were then dispersed into the PAA/laponite mixture. Cancer cells produced more H_2_O_2_ and glutathione (GSH) than normal cells. These molecules can break down manganese dioxide (MnO_2_) through a process called redox-mediated decomposition. This affected the electrochemical sensing properties of hydrogels that contain MnO_2_. When MnO_2_-based hydrogels were treated with different types of cancer cells (such as PC-3, HeLa, and B16F10) and normal cells (CHO-K1) in vitro, the cancer cells enhanced the tunability of the electrochemical sensing, while the normal cells had minimal effects.

Conductive polymer hydrogels (CPHs) with excellent electrical properties, mechanical flexibility, biocompatibility, and ease of processing have been increasingly used for biosensing systems. CPH-based biosensors display rapid response, notable sensitivity, low detection limit, and broad linear range, which has great potential for cancer detection. However, CPHs as new materials in biomedicine, and their potential is not fully explored. One of the challenges is how to sterilize them without damaging their structure and function. It was reported that common sterilization methods can decrease the quality of CPHs.^98^ Moreover, CPHs exhibit limited electrostability and electroactivity when compared to metals. They only display metallic conductivity when doped with suitable dopants or made into composites with foreign materials.^99^ Furthermore, CPH-based biosensors need to be highly responsive and distinguish tiny human motions when they are used in practical applications in human health care.^82^ Improving performance through the design, modification, and assembly of fluorescent, ion-conducting, and conductive hydrogels will potentially address these issues. Very recently, a porosity-tunable electroconductive hydrogel sensor has been designed by incorporating diselenide-cross-linked carbon dot (dsCD)-loaded zwitterionic polymer dots (PDs) into the hydrogel for cancer detection.^100^ The dsCD@PD hydrogel sensor showed changes in pore size owing to diselenide bond cleavage in dsCDs and release of zwitterionic PDs in the cancerous environment (e.g., higher ROS and acidic pH of cancer cells). This led to distinct conductivity and negative gauge factor of dsCDs@PD hydrogel in the presence of cancer cells and positive gauge factor in the presence of normal cells.^100^

### 3.4 Cell-Based Hydrogel Biosensor

Cell-based biosensors are novel detection tools that utilize the cells’ innate ability to sense and respond to environmental stimuli. By engineering their biosensing mechanisms, cell-based biosensors can detect specific molecules of interest and generate measurable signals. Cell-based biosensors have many advantages over conventional detection methods, such as mobility, low cost, modularity, stability, operational convenience, and controllability.^101^ Moreover, cell-based biosensors have a flexible design and output that can be customized according to the application-specific requirements. Cell-based biosensors can also perform rapid and sensitive analysis for in situ monitoring within cells in addition to sensing and detecting the target analyte.^102^ Therefore, cell-based biosensors have demonstrated great potential in various areas of applications, such as bioproduction,^103^ environmental monitoring,^104^ and biomedical diagnostics.^105^

Cell-based biosensors are emerging as powerful tools for cancer detection.^106^ For instance, Chien et al. engineered bacteria with controllable genetic circuits that can sense pH, oxygen, or lactate via the control of the essential gene expression for therapy.^107^ The biosensor was constructed by synthesizing promoters from Integrated DNA Technologies (IDT) and native promoters including pPepT, pLldR, and pCadC from *E. coli* Nissle 1917, which were cloned in front of the *sfGFP* gene of ColE1 pTD103 sfGFP plasmid. To tune the sensitivity of the genetic circuit, different gene copy numbers, RBSs, antisense promoters, and protein degradation tags were designed and installed using Gibson assembly. The biosensors were further integrated into the bacterial genome. These bacteria-carrying biosensors, connected by an AND gate, increased tumor specificity in mice with subcutaneous tumors. Some bacterial species can selectively colonize primary and metastatic tumors. In addition, synthetic biology approaches have helped design delivery systems that target tumor-specific biomarkers.^108,109^ The engineered genetic circuits for specific bacteria can be optimized to improve cell-based cancer detection.

**Figure 6:**
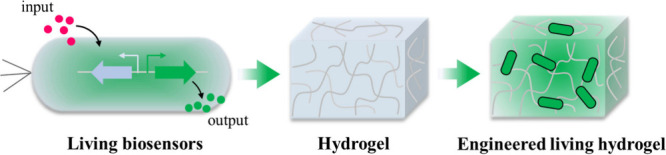
Combination of living biosensors and hydrogel to generate engineered living hydrogel.

Commercializing cell-based biosensors is challenging due to several obstacles in their construction and application for sensing target analytes. The construction process of a cellular biosensor requires a special system and a lot of time to optimize the initial constructs and improve the sensing performances, such as sensitivity and dynamic range. Synthetic biology offers various strategies, such as promoter engineering, ribosome binding site (RBS) engineering, and directed evolution, to improve the performance in vitro and in vivo. However, very few tumor-targeting bacteria have reached the clinical stage, because living bacteria cannot be sterilized by heating in contrast to small molecules or other nonviable analytical agents. This poses a major challenge to the principles of good manufacturing practices (GMP) and raises biosafety and biosecurity concerns. Hydrogels could solve these issues by providing a stable and immobilized space for cell-based biosensors. In recent years, engineered living materials have been designed that contain living cells with responsive function and polymeric matrices (e.g., hydrogels)^110,111^ (Figure 6). Engineered living hydrogels consist of two components (living cells and hydrogels) that can be programmed to meet specific targets. The living cells are genetically engineered to perform various functions, such as biosensing, while the hydrogels provide spatial distribution and mechanical confinement for them. Hypothetically, when analytes (e.g., molecular biomarkers) enter hydrogel space, they can be recognized by the sensing module which is the signal transducer (e.g., transcription factors) responsible for the recognition of the analytes and transduction of this signal to the reporter module. The reporter module then generates a measurable signal output (e.g., light, color, or fluorescence changes). Furthermore, by engineering and optimizing the function of living cells and the networks of the nonliving matrix of the hydrogel, the engineered living hydrogels can sense and respond to small changes in the local environment, enabling their applications in disease treatment, environmental remediation, and cancer treatment. Weiden et al. have commented that hydrogel scaffolds loaded with activated T cells can be used for antitumor immunotherapy.^112^ While cell-free hydrogel biosensors have been extensively explored for cancer detection, cell-based hydrogels represent an emerging area with a limited body of existing literature. That is why this section is relatively thin compared to cell-free hydrogel biosensors. This necessitates further research to fully harness their potential in this field. Overall, with the advances in bioengineering and advanced manufacturing technologies, the integration of living cells or constructs with hydrogel can be expected to be used in more fields, achieving unprecedented performance and functionality.

## 4 Conclusions and Outlook

In the dynamic landscape of cancer research, novel drugs and therapies continue to emerge, offering hope for improving treatment outcomes. However, the pace of technological advancements in early diagnosis and screening remains sluggish. To bridge this gap, there is a pressing need for highly efficient biosensors capable of swiftly analyzing cellular changes that can detect early stage cancer or identify postoperative cancer recurrence. Hydrogel-based biosensors have emerged as promising tools in the realm of cancer detection and monitoring. Their biochemical and biophysical properties can be easily tuned, which lends them the ability to detect diverse tumor cells and biomarkers at low detection limits and across broad ranges. Hydrogel-based biosensors hold great promise for medical applications, but several challenges hinder their widespread adoption. These challenges include (1) insufficient mechanical strength affecting their durability, long-term reliability, and consistent performance; (2) the intricate nature of hydrogels can make them challenging to manipulate during fabrication and use; and (3) ensuring stable storage conditions and reproducible results remain an issue. Furthermore, it is also important to develop compact and miniaturized hydrogel-based biosensors that can generate signals to be readily collected by portable devices (e.g., smartphones). This will make them potential devices for cancer point-of-care diagnostics. For cancer detection, hydrogel-based biosensors are currently more commonly used ex vivo rather than in vivo. Ex vivo testing minimizes potential risks associated with implantation, such as infection, and avoids complications from the body’s immune response or interference from other biological processes. This controlled environment also facilitates the accurate detection of cancer biomarkers. Looking ahead, in vivo hydrogel sensors hold significant promise. These sensors could be strategically placed near high-risk areas or organs to enable early detection of initial cancer signs, potentially leading to more timely diagnosis and treatment. Furthermore, research is ongoing for theragnostic applications, where the sensor not only detects cancer cells but also delivers targeted therapy, offering a more comprehensive approach. While further research is needed to address biocompatibility, stability, and signal transmission challenges before widespread clinical use of in vivo sensors can be realized, hydrogel-based biosensors have the potential to revolutionize early cancer diagnosis in the coming decade. By overcoming these hurdles, we aim to unlock the potential for timely, accurate detection, ultimately saving lives and improving patient outcomes worldwide.
